# Clinical, histopathological, and molecular characterization of *Mycoplasma* species in sheep and goats in Egypt

**DOI:** 10.14202/vetworld.2021.2561-2567

**Published:** 2021-09-28

**Authors:** Walid S. Mousa, Ahmed A. Zaghawa, Ahmed M. Elsify, Mohamed A. Nayel, Zarroug H. Ibrahim, Khalid A. Al-Kheraije, Hesham R. Elhalafawy, Dina El-Shafey, Anis Anis, Akram A. Salama

**Affiliations:** 1 Department of Animal Medicine and Infectious Diseases, Faculty of Veterinary Medicine, University of Sadat City, Egypt; 2 Department of Veterinary Medicine, College of Agriculture and Veterinary Medicine, Qassim University, Buraydah, Saudi Arabia; 3 Department of Biomedical Sciences, College Veterinary Medicine, Sudan University of Science and Technology, Khartoum, Sudan; 4 Department of Mycoplasma, Animal Health Research Institute, Dokki, Giza, Egypt; 5 Department of Pathology, Faculty of Veterinary Medicine, University of Sadat City, Egypt.

**Keywords:** goats, minimum inhibitory concentration, *Mycoplasma*, polymerase chain reaction, prevalence, sheep

## Abstract

**Background and Aim:**

*Mycoplasma* infection in small ruminants is a serious problem in sheep and goat herds around the world. It is responsible for high economic losses and decreased animal productivity. This study aimed to highlight the clinical, histopathological, minimum inhibitory concentration (MIC), and molecular characterization of *Mycoplasma* species in sheep and goats in Menoufiya Governorate, Egypt.

**Materials and Methods:**

A total of 234 samples were collected; 104 samples were collected from pneumonic lung tissues from the abattoir, in addition, 10 and 20 samples collected from apparently and diseased sheep, respectively, and 40 and 60 samples were collected from apparently and diseased goats, respectively, which were subjected to isolation onto pleuropneumonia-like organism medium. Polymerase chain reaction (PCR), histopathological examination, and determination of the MIC were also performed.

**Results:**

Of 104 samples of lung tissues showing pneumonic lesions, 56 (53.84%) were positive for *Mycoplasma* isolation. The positive isolation of *Mycoplasma* from 10 and 20 samples from apparently and diseased sheep was 30% and 40%, respectively as well as the positive isolation of *Mycoplasma* wa*s* 17% and 56.66% out of 40 and 60 apparently healthy and diseased field goat’s cases, respectively. All the diseased sheep and goats showed respiratory manifestations, including cough, bilateral nasal discharge, conjunctivitis, and systemic reaction. Evaluation of the MIC for *Mycoplasma* *ovipneumoniae* revealed that lincospectin and tylosin were the most effective antibiotics at 2.5 mg/mL. Histopathological examination of affected lung tissue showed extensive hemorrhagic pneumonia with extensive alveolar hemorrhage. The PCR technique proved to be a rapid, specific, and sensitive method for the detection of *M. ovipneumoniae* and *Mycoplasma arginini* at 390 and 326 bp, respectively.

**Conclusion:**

*M. ovipneumoniae* and *M. arginini* were the most prevalent species associated with respiratory infections in sheep and goats in the study area. Further studies are needed to investigate the role of these species in dissemination of the disease within herds of small ruminants.

## Introduction

Respiratory syndromes are commonly encountered in sheep and goat populations. They are often caused by multifactorial agents, including infectious agents, such as viruses, bacteria, fungi, and parasites, as well as predisposing management factors, such as stress and climatic factors that lead to significant losses [1,2]. Numerous *Mycoplasm*a serotypes are associated with various pathological complications in small ruminants, including respiratory signs, causing major losses, especially in African countries and Egypt [3,4]. *Mycoplasma* belong to the class Mollicutes, which contains eight genera, of which five are found in animals: *Mycoplasma, Ureaplasma*, *Acholeplasma*, *Anaeroplasma*, and *Asteroplasma*. *Mycoplasma* and *Ureaplasma* are more pathogenic in animals [5]. Indirect economic losses and infertility, high morbidity, and occasionally mortality are associated with acute/subacute or chronic pneumonic *Mycoplasma* infection [6]. Outbreaks of infection by virulent strains of *Mycoplasma ovipneumoniae* often occur in lambs from different flocks housed together [7], usually associated with heavy rain, animal transportation, poor climatic conditions, and introduction of infected animals into susceptible herds [8]. Secretions from diseased or carrier animals have a substantial role in the maintenance and spread of the disease among herds through inhalation of infected droplets from animals in close contact [9,10]. Postmortem and histopathological examination [11] reveals gray or red areas of consolidation in the affected lungs, with marked pleuritis and pleural effusion of yellowish fluid, and fine granular texture with hepatization in cross-sections of affected surfaces.

Difficulties in the diagnosis of *Mycoplasma* infection by traditional biochemical and serological tests, due to the fastidious nature of *Mycoplasma* species, have encouraged researchers to develop modern molecular techniques for rapid and effective diagnosis of *Mycoplasma* infection, such as polymerase chain reaction (PCR) [10,12]. PCR is a valuable, rapid, recent molecular approach for the diagnosis of *Mycoplasma* infection and genotyping of *Mycoplasma* species [13].

This study emphasizes that *M. ovipneumoniae* and *Mycoplasma arginini* were the most prevalent species associated with respiratory infections in sheep and goats in Giza and El-Menoufiya Governorate, Egypt, as well as reporting the crucial role of PCR for rapid and specific detection of *Mycoplasma* species. In addition, this study highlights the devastating effects of *Mycoplasma* species on lung tissue, which shows extensive hemorrhagic pneumonia with extensive alveolar hemorrhage.

This study aimed to determine the prevalence of *Mycoplasma* species in sheep and goats in Egypt, with molecular detection of the most prevalent species. In addition, it evaluated the minimum inhibitory concentration (MIC) of different antibiotics against the obtained species, as well as performing histopathological examination.

## Materials and Methods

### Ethical approval

This study followed the guidelines of the Ethics Committee and current legislation on research and ethical approval of the Faculty of Veterinary Medicine (approval no. VUSC-014-2-21), University of Sadat City, Egypt.

### Study period, sampling, and clinical examination

The study was conducted from November 2017 to April 2018. A total of 104 samples of lung tissues from rams showing pneumonic lesions were collected from El-Basateen abattoir, Giza Governorate, Egypt. In addition, nasal swabs from 30 sheep (10 apparently healthy and 20 diseased) and 100 goats (40 apparently healthy and 60 diseased) were collected from El-Menoufiya Governorate, Egypt. All examined sheep and goats in the field condition showed respiratory manifestations, including bilateral nasal discharge, cough, conjunctivitis, and fever. The samples were transported to the laboratory under cold conditions (4°C) for bacteriological examination.

### Isolation and identification of Mycoplasma

The collected samples were cultivated in pleuropneumonia-like organism (PPLO) broth for 3 days, then cultured into PPLO agar medium for another 3 days at 37°C, then examined with a stereo microscope every 2 or 3 days. If the characteristic mycoplasmal “fried egg” colonies appeared on the agar plates, agar blocks with *Mycoplasma* colonies were transferred into broth medium and incubated at 37°C for 2 or 3 days and then subjected to purification. *Mycoplasma* species were identified by a digitonin sensitivity disk, and biochemical characterization was performed by a glucose fermentation test and arginine deamination test, according to Valsala *et al*. [14].

### MIC

The MIC was determined in a representative field strain *(M. ovipneumoniae*) for seven antibiotics: danofloxacin 25%, Draxxin 10%, florfenicol 30%, lincospectin 100/50, oxytetracycline 5%, streptomycin 100%, and tylosin 100%. *M. ovipneumoniae* was sensitive to lincospectin (0.5 mg/mL) and tylosin (0.5 mg/mL). The MIC was determined according to Hannan [15] in 96-well microtiter plates with wells containing growth control (broth medium without antibiotic), sterility control (broth medium without antibiotic and *Mycoplasma* inoculum), and pH control (broth medium adjusted to pH 6.8). *Mycoplasma* broth medium (pH 7.8) was supplemented with 0.5% (w/v) sodium pyruvate, 0.5% (w/v) glucose, and 0.005% (w/v) phenol red. The MIC value of each isolate was defined as the lowest concentration of the antibiotic that completely inhibited growth in the broth (no pH and color change) after 1 week. Briefly, 2-fold dilutions were prepared in the range of 0.039-10 mg/mL for fluoroquinolones, 0.125-32 mg/mL for florfenicol, 0.25-64 mg/mL for gentamicin and tetracyclines, 0.5-128 mg/mL for macrolides, and 1-256 mg/mL for lincospectin.

### Histopathological examination

Lung tissue samples were fixed in 10% neutral buffered formalin (pH 7.4) for 72 h, washed, dehydrated, embedded in paraffin wax, serially sectioned with a microtome at 3 mm thickness, and stained with hematoxylin and eosin for histopathological investigation. Leica DMLB microscopes (Leica Microsystems Wetzlar GmbH Ernst-Leitz-Strasse D-35578 Wetzlar Germany) were used in this study. Histological photographs were taken with a Leica EC3 digital camera as described by Wäsle *et al*. [16].

### PCR for molecular detection of Mycoplasma strains

DNA extraction was performed with the GF-1 Tissue DNA Extraction Kit (Vivantis), according to the manufacturer’s instructions. The PCR reaction was performed in a volume of 50 mL, including 25 mL My Taq Red Mix, 2×, 1 mL from each primer (20 mM of each), DNA template 200 ng, and completed with sterile water up to 50 mL. Common primer 16S RNA gene and specific primers (16-23 S intergenic spacer) were used for molecular detection of *M. ovipneumoniae* and *M. arginini*. The PCR cycle conditions and references for molecular diagnosis of *M. ovipneumoniae* and *M. arginini* are listed in Table-1 [17-19].

**Table-1:** Primers sequence, PCR cycling conditions for molecular detection of *Mycoplasma ovipneumoniae* and *Mycoplasma arginine.*

Strain	Primer sequence	Fragment size (bp)	Primary denaturation	30-35 cycle				Reference

				Secondary denaturation	Annealing	Extension	Final extension	
*Mycoplasma species (16SRNA)*	F: AGA CTC CTA CGG GAG GCA GCA R: ACT AGC GAT TCC GAC TTC ATG	1000	94°C 5 min	95°C 1 min	55°C 45 s	72°C 1 min	72°C 10 mi	[17]
*Mycoplasma ovipneumoniae* 16S-23-RNA	F:GGAACACCTCCTTTCTACGG’ R:CCAAGGCATCCACCAAATAC	390	95°C 15 min	95°C 30 s	58°C 30 s	72°C 30 s	72°C 5 min	[18]
*Mycoplasma arginine* 16S-23-RNA	F: TGA TCA TTA GTC GGT GGA GAG TTC R: TAT CTC TAG AGT CCT CGA CAT GAC TC	326	94°C 3 min	94°C 1 min	60°C 30 s	72°C 60 s	72°C 4 min	[19]

## Results

### Prevalence and bacteriological examination of Mycoplasma in lung tissues and nasal swabs from sheep and goats

**Figure-1:**
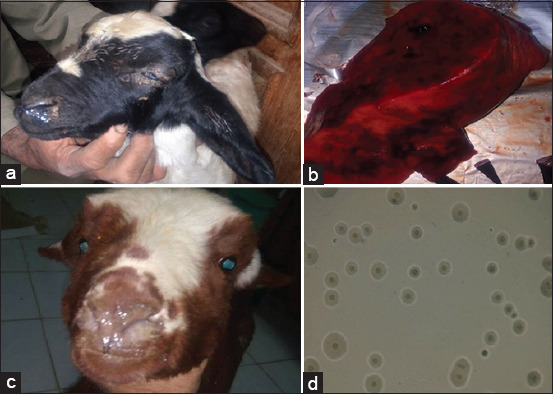
(a) Lamb (3 months old) showed unilateral nasal discharges and ocular discharge with depression. (b) Kid (3 months old) showed bilateral mucopurulent nasal discharges. (c) Lung tissue of a 3-year-old ram showed reddening, consolidation, and localized necrosis in different areas of the lung. (d) Fried egg colonies of mycoplasma using Stereo microscopes.

Of 104 samples of lung tissues collected from rams at the abattoir, 56 (35.6%) were positive for *Mycoplasma* isolated into PPLO-specific medium. There were 3 and 8 sheep cases positive for *Mycoplasma* out of 10 and 20 apparently healthy and diseased sheep in field cases respectively. On the other hand, 7 and 34 goats cases were positive for *Mycoplasma* out of 40 and 60 field apparently and diseased goat cases, respectively (Table-2). Clinical examination of the diseased cases in sheep and goats showed respiratory manifestations, including cough, bilateral nasal discharge, conjunctivitis, and systemic reaction (fever) (Figures-1a and b). In postmortem examination, the pneumonic lung tissues showed reddening, consolidation, and localized necrosis in different areas of the lung (Figure-1c). *Mycoplasma* in PPLO medium typically appears as “fried egg” colonies (Figure-1d).

**Table-2:** Results of bacteriological examination of *Mycoplasma* from lung tissues and nasal swabs collected from sheep and goats.

	Rams	Sheep		Goats		Total samples
		
	Lung tissue	Apparently healthy	Diseased	Apparently healthy	Diseased	
No of examined animals	104	10	20	40	60	234
Positive isolation	56	3	8	7	34	108
% of isolation	56.8	30%	40%	17.5%	56.66%	46.2%

### Evaluation of the MIC against field M. ovipneumoniae strain

The MIC was determined in representative field strains of *M. ovipneumoniae* against seven antibiotics: danofloxacin, Draxxin, florfenicol, lincospectin, oxytetracycline, streptomycin, and tylosin. The results showed that *M. ovipneumoniae* isolates were more sensitive to lincospectin at a concentration of 100/50 and tylosin 100% *in vitro*. Resistance was observed for the other antibiotics.

### Histopathological findings in sheep and goat lungs infected by Mycoplasma species

**Figure-2:**
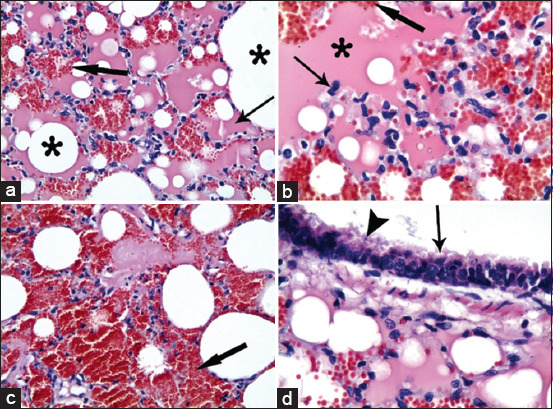
Lung, sheep. Mycoplasma-positive samples in acute stage of pneumonia: (a) A widespread homogenous eosinophilic inflammatory exudate inside the alveoli (thin arrow) and alveolar hemorrhage (thick arrow). Alveoli (asterisk). (b) Active alveolar macrophages (thin arrow) and alveolar hemorrhages (thick arrow) in homogenous eosinophilic inflammatory exudate inside the alveoli (asterisk). (c) Extensive hemorrhagic pneumonia (thick arrow). (d) Swelling and hydropic degeneration (arrow) and necrosis of epithelium lining of the bronchioles. H and E stain, a and c ×10; b and d ×40.

**Figure-3:**
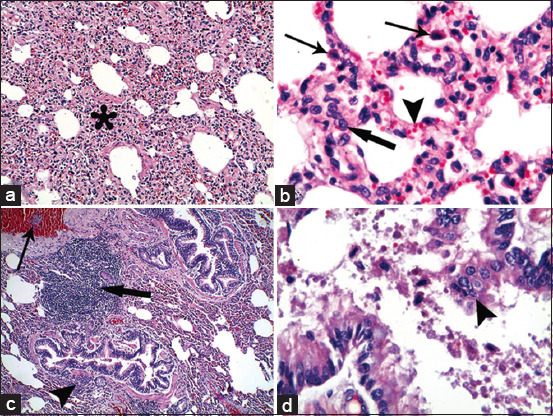
Lung. (a and b) Mycoplasma-positive sample in subacute of pneumonia from goat lung tissues: (a) Subacute interstitial pneumonia (asterisk). (b) High magnification from figure. (a) Active alveolar macrophages (thin arrows) and thick interalveolar septa by mononuclear cell infiltration (thick arrow) and alveolar capillary dilatation (arrowhead). (c and d) Mycoplasma-positive sample in chronic stage of pneumonia from sheep lung tissues: (C) Multifocal nodules of mononuclear cell infiltration in lung tissues (thick arrow), mononuclear cells aggregate in bold stream (thin arrow) and peribronchiolar lymphoid cell infiltration (arrowhead). (d) Desquamation of necrotic epithelial cells of the bronchioles inside its lumen (arrowhead). H and E stain, a ×10; c ×4; b and d ×40.

In the acute stage of pneumonia in ­*Mycoplasma*-positive samples, sheep lung tissues showed a widespread homogenous eosinophilic inflammatory exudate inside the alveoli with alveolar hemorrhages and marked active alveolar macrophages (Figures-2a and b). In addition, extensive hemorrhagic pneumonia was detected in some cases (Figure-2c), and hydropic degeneration and/or necrosis of the epithelial lining of the bronchioles were recorded (Figure-2d). In the subacute stage of pneumonia in *Mycoplasma*-positive samples, goat lung tissues showed interstitial pneumonia, active alveolar macrophages, thick interalveolar septa by mononuclear cell infiltration, and alveolar-capillary dilatation (Figures-3a and b). In the chronic stage of pneumonia in *Mycoplasma*-positive samples, sheep lung tissues showed multifocal nodules of mononuclear cell infiltration, mononuclear cells aggregated in the bloodstream, peribronchiolar lymphoid cell infiltration, and desquamation of necrotic epithelial cells of the bronchioles inside the lumen (Figures-3c and d).

### Molecular identification of Mycoplasma species in sheep and goats by PCR

**Figure-4:**
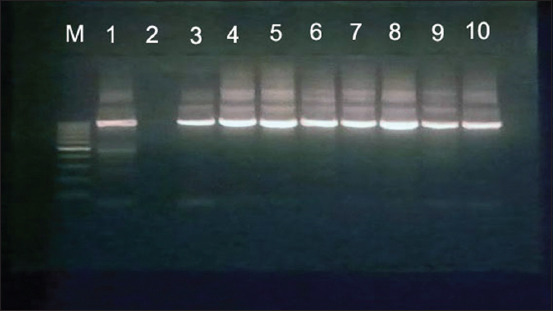
1.5% agarose gel showing PCR product of *Mycoplasma* species using *16S rRNA* primer gene for mycoplasma at 1000 bp. M: DNA marker, lane 1: Control +ve, lane 2: Control –ve, lane 3-10: +ve samples.

**Figure-5:**
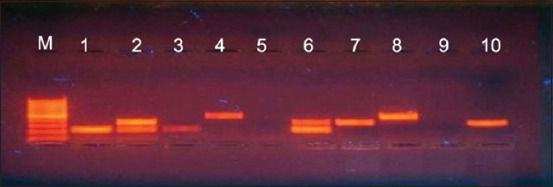
1.5% agarose gel showing PCR product of *Mycoplasma ovipneumoniae* at 390 bp using (16S-23S intergenic spacer). M showing the 100 bp-1 kb DNA ladder. Lanes 2, 6, 7, and 10: Positive samples, lanes 1, 3, 4, 5, and 8: Negative samples.

**Figure-6:**
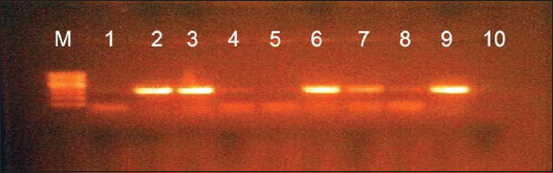
1.5% agarose gel showing PCR product of *Mycoplasma arginini* using specific primer (16S-23S intergenic spacer) at 326 bp. M showing the 100 bp-1 Kb DNA ladder. Lanes 1, 4, 5, and 8: −ve *M. arginine* while lanes 2, 3, 6, 7, and 9: +ve M. arginine.

The identification of *Mycoplasma* species recovered from sheep and goats in this study was an efficient tool for the detection of *Mycoplasma* species at 1000 bp using common universal 16S rRNA primer, as shown in Figure-4. The molecular identification of *M. ovipneumoniae* was successfully amplified using 16S-23S intergenic spacer gene at 390 bp (Figure-5). *M. arginini* was molecularly identified by a specific primer in which the amplified band was detected at 326 bp (Figure-6).

## Discussion

Respiratory infections are responsible for great economic losses in small ruminants [20]. Although many etiological agents are involved, *Mycoplasma* species are considered a particularly substantial cause of such infections and exert a significant socioeconomic effect, particularly in areas where small ruminants are an important source of milk and meat [4]. Various serious problems are associated with *Mycoplasma* infection, such as contagious caprine pleuropneumonia, conjunctivitis, arthritis, mastitis, and mild respiratory distress [21]. *M. ovipneumoniae* and *M. arginini* are frequently present in pneumonic lesions among small ruminants [14,22].

In the present study, 30 sheep and 100 goats were clinically examined. Examination of diseased sheep and goats revealed fever, anorexia, depression, bilateral mucopurulent nasal discharge, lacrimation, and mouth breathing in most cases, as well as cough with the expulsion of nasal discharge. These results are in agreement with those of Awan *et al*. [11], who reported marked fever, watery to thick nasal discharge, and difficult breathing in goats with mycoplasmosis, as well as signs of pain in some goats when the chest was touched. Ayling and Nicholas [7] and Dezfouli *et al*. [23] demonstrated that clinical signs of mycoplasmosis in lambs showed increased respiratory rates that might be complicated with other infections and ended by death. In the present study, 104 samples of ram lung tissues collected from the abattoir showed lesions of pneumonia, including reddening, consolidation, and localized necrosis in different areas of the lung. Fibrinopurulent membrane on the pleural surface and serofibrinous fluid were observed in the thoracic and abdominal cavities. These results agree with those of Sheikh *et al*. [21], and Yatoo and Kanwar [8] reported that the most characteristic postmortem lesions of mycoplasmosis include reddening, consolidation, and purulent focal and localized necrosis, in addition to fibrinopurulent membrane on the pleural surface and serofibrinous fluid in the thoracic and abdominal cavities.

In the current study, of a total of 234 samples (104 samples of pneumonic lung tissues, 30 samples from diseased and apparent healthy sheep, and 100 samples from diseased and apparent healthy goats), 108 (46.2%) were positive in bacterial isolation. A similar finding was reported by Abdou [24], who reported a 42.5% prevalence rate of *Mycoplasma*. On the other hand, prevalence rates of *Mycoplasma* were 40% and 17.85% in apparently healthy sheep and goats respectively in Egypt [25]. Mostafa [26] reported that the prevalence rates of *Mycoplasma* in apparently healthy sheep and goats were 14.67% and 20.39%, respectively. The higher prevalence rates of *Mycoplasma* in our survey may be due to the bad hygienic measures applied in animal management and husbandry practices.

With regard to the use of the MIC as the reference point for comparison to determine the efficacy of antibiotics [15], in our study, the MIC for representative field strains of *M. ovipneumoniae* using seven different antibiotics showed that *M. ovipneumoniae* was more susceptible to lincospectin and tylosin and was resistant to other antibiotics. This result was supported by Al-Momani *et al*. [27] and Tatay-Dualde [28] who showed that tylosin, erythromycin, and lincosamides were the most effective antibiotics against *Mycoplasma* species. On the other hand, an earlier study by Otlu [29] reported that *Mycoplasma* species were sensitive to enrofloxacin and resistant to streptomycin. Furthermore, Eissa *et al*. [30] reported that enrofloxacin was effective against *M. ovipneumoniae* isolates due to its wide spectrum of activity, lipid solubility, and weakly basic reaction.

The histopathological examination of 10 randomly selected samples that were positive for *Mycoplasma* isolation showed extensive hemorrhagic pneumonia with eosinophilic exudate and extensive alveolar hemorrhage with infiltration of mononuclear cells. In addition, degeneration and deciliation of the surface epithelium of bronchiolar mucosa were observed during the histopathological examination. This was previously described by Adehan *et al*. [6], who reported that most alveoli and bronchioles were filled with a mixture of neutrophils and macrophages, whereas other alveoli were filled with edema fluid and fibrin. In addition, Hernandez *et al*. [9] observed necrotizing vasculitis in vessel walls, with infiltration by inflammatory cells and thrombus formation.

The definitive diagnosis of *Mycoplasma* infection is based on typical isolation into a specific medium, which is time-consuming and requires special procedures. Molecular approaches, such as PCR, are rapid, specific, and accurate for diagnosis of infection by *Mycoplasma* species, as shown by Amores *et al*. [10] and Settypalli *et al*. [31], by targeting specific genes [32]. In addition, Besser *et al*. [33] successfully detected *M. ovipneumoniae* recovered from bronchoalveolar lavage fluid in sheep based on 16S rRNA species-specific gene.

In the current study, *M. ovipneumoniae* was detected with a prevalence of 3.8% (4/104) and *M. arginini* was detected with a prevalence of 4.8% (5/104) in lung tissues of sheep. Similar findings in Egypt were reported by Abdel-Halium *et al*. [3], who identified both *M. arginini* and *M. ovipneumoniae* from sheep and goats with pneumonic lesions. On the other hand, higher prevalence rates were reported in Nigeria [34] *M. ovipneumoniae* and *M. arginini* were detected with prevalence rates of 61.5% and 30.8%, respectively, in lung tissues of sheep. In addition, in Benin [6], *M*. *ovipneumoniae* and *M. arginini* were detected with prevalence rates of 44.4% and 11.1%, respectively, in lung tissues of sheep. In a comparative study in Turkey [35] *M. ovipneumoniae* and *M. arginine* were detected with prevalence rates of 65% and 35%, respectively. Rekha *et al*. [36] detected only *M. arginini* in sheep with caprine pneumonia in India.

## Conclusion

*M. ovipneumoniae* and *M. arginini* are the most common *Mycoplasma* species in sheep and goats with respiratory infections in Giza and El-Menoufiya Governorates in Egypt. Lincospectin and tylosin are the most effective antibiotics for the treatment of *Mycoplasma* infection in small ruminants. PCR is an effective method of detection of *Mycoplasma* species. Histopathological examination shows the devastating effects of *Mycoplasma* infection in lung tissue, including extensive hemorrhagic pneumonia and alveolar hemorrhage with degeneration and deciliation of the surface epithelium of the bronchiolar mucosa. Further studies are needed for a better epidemiological picture of disease dissemination by *Mycoplasma* species in small ruminants in Egypt.

## Authors’ Contributions

WSM, AAZ, AAS, MAN, AME, AAS, and HRE: Involved in the conception of the research idea and methodology design, performed the data analysis and interpretation, and prepared the manuscript for publication, HRE, DE, ZHI, and KAA: Participated in the design of the methodology and involved in laboratory work, and AA: Participated in the histopathology work and data analysis and contributed their scientific advice during the work and revision. All authors read and approved the final manuscript.
